# Rapamycin preserves gut homeostasis during *Drosophila* aging

**DOI:** 10.18632/oncotarget.5895

**Published:** 2015-09-29

**Authors:** Xiaolan Fan, Qing Liang, Ting Lian, Qi Wu, Uma Gaur, Diyan Li, Deying Yang, Xueping Mao, Zhihua Jin, Ying Li, Mingyao Yang

**Affiliations:** ^1^ Animal Genetic Resources Exploration and Innovation Key Laboratory of Sichuan Province, Sichuan Agricultural University, Chengdu, P.R. China; ^2^ School of Biotechnology and Chemical Engineering, Ningbo Institute of Technology, Zhejiang University, Zhejiang, P.R. China

**Keywords:** Drosophila, gut homeostasis, intestinal stem cell, rapamycin, aging, Gerotarget

## Abstract

Gut homeostasis plays an important role in maintaining the overall body health during aging. Rapamycin, a specific inhibitor of mTOR, exerts prolongevity effects in evolutionarily diverse species. However, its impact on the intestinal homeostasis remains poorly understood. Here, we demonstrate that rapamycin can slow down the proliferation rate of intestinal stem cells (ISCs) in the aging guts and induce autophagy in the intestinal epithelium in *Drosophila*. Rapamycin can also significantly affect the FOXO associated genes in intestine and up-regulate the negative regulators of IMD/Rel pathway, consequently delaying the microbial expansion in the aging guts. Collectively, these findings reveal that rapamycin can delay the intestinal aging by inhibiting mTOR and thus keeping stem cell proliferation in check. These results will further explain the mechanism of healthspan and lifespan extension by rapamycin in *Drosophila.*

## INTRODUTION

Lifespan in many organisms can be extended by improving genetic factors, for example reducing activity of the mechanistic target of rapamycin (mTOR) [1] and Insulin/IGF1 signaling (IIS) [2-4], as well as environmental conditions, such as dietary restriction [5-7] and decreasing oxidative stress [8,9]. The different mechanisms are likely to act through related processes, particularly by increasing stress-protective gene expression in differentiated somatic cells, prolonging their functional lifespan and delaying tissue deterioration [10-12]. In addition to such stress protective mechanisms, metazoans also maintain tissue homeostasis through regenerative processes that mainly rely on the long-term maintenance of a functional population of somatic stem and progenitor cells. For these cells, relationship between stress protection and lifespan is expected, as their long-term maintenance is critical to conserve regenerative capacity [13].

In recent years, maintenance of intestinal homeostasis is shown to play a key role in lifespan determination in *Drosophila* [13-15]. Tissue homeostasis in the midgut is maintained by multipotent intestinal stem cells (ISCs), which are distributed along the basement membrane [16-18]. Division of an ISC gives rise to one daughter cell that retains stem cell fate and another daughter cell that becomes an enteroblast (EB), both expressing a transcription factor called Escargot (*esg*). Thus, expression of *esg* is often used as a surrogate marker for ISCs and EBs. After ISC division, the daughter EB does not divide again and differentiates into either a large, polyploid enterocyte (EC) or a small, diploid entero-endocrine (ee) cell. In response to stress conditions, however, ISC proliferation is strongly increased, a regenerative response that allows restoring large parts of the intestinal epithelium in response to damaging agents, such as pathogens, genotoxins, or ROS inducing compounds [19-21]. This regenerative function of ISCs have deleterious consequences for the organism, as excessive proliferation in response to stress is accompanied by the accumulation of mis-differentiated cells in the intestine, which ultimately disrupts epithelial integrity with a dysplastic phenotype [22]. In the aging gut, such dysplasia is widely observed under normal culture conditions, suggesting that an age-related over-proliferation of ISCs contribute to the loss of intestinal function and to the increased mortality of aged flies [23, 24]. ISC self-renewal and differentiation is controlled by the Notch and mTOR signaling pathways. The long-term stem cell maintenance is further ensured by mechanisms that prevent activation of mTOR signaling [25-27].

Rapamycin is the most specific TOR inhibitor known and it acts through association with the intracellular protein FKBP12, which binds to the FKBP12-rapamycin-binding (FRB) domain of TOR, inhibiting TORC1 activity. Although rapamycin does not bind to the catalytic domain of TOR, it reduces phosphorylation of two downstream TORC1 targets, S6K and 4E-BP [1]. The effect of rapamycin on lifespan extension has been studied in many species but its effect on gut homeostasis is not fully elucidated.

In order to examine whether rapamycin can preserve gut homeostasis during aging, we used *Drosophila* intestine as an accessible model system. Our results revealed a significant correlation between rapamycin intake and slowing down of the intestinal aging. Most importantly, we showed that rapamycin limits the proliferation rates of intestinal stem cells by moderately inhibiting mTOR leading to delay in the microbial expansion during gut aging. Our findings demonstrate that maintenance of the guts homeostasis during aging could be one of the important effect of rapamycin that extends lifespan in *Drosophila*.

## RESULTS

### Rapamycin slows ISCs proliferation rate and extends lifespan

**Figure 1:**
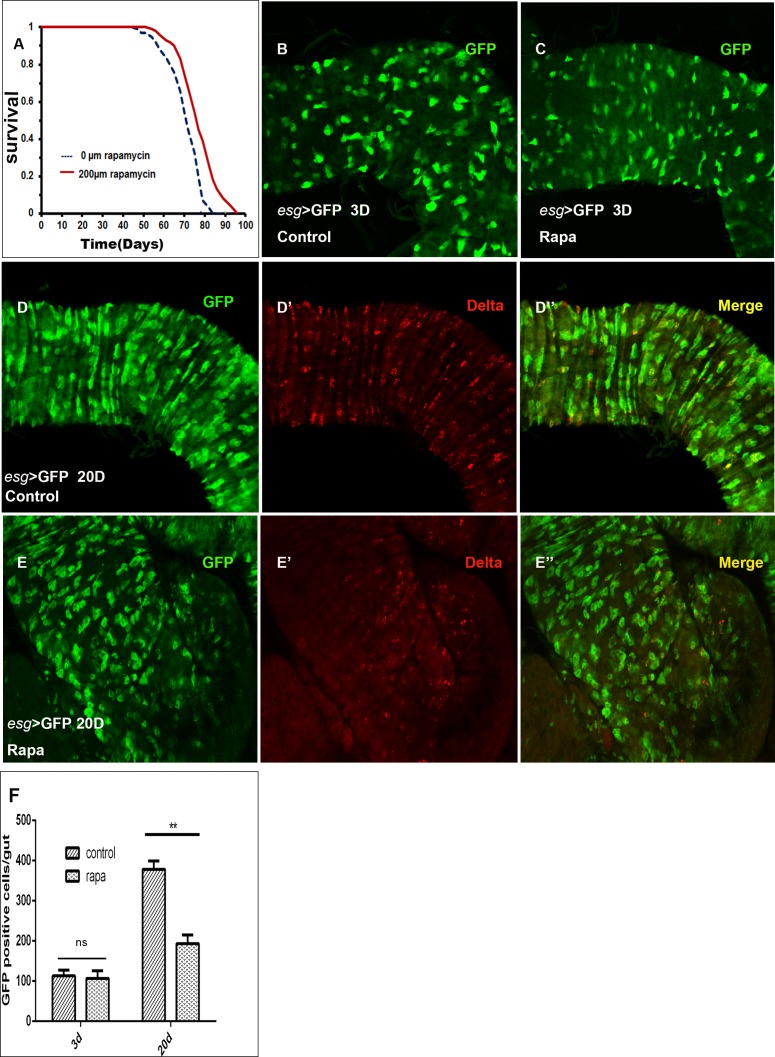
Rapamycin slows ISCs proliferation rates **A.,** Rapamycin treatment extends the life span of w^Dah^ females. Compared to flies on control food (0μM of rapamycin), flies on food having 200μM of rapamycin have increased median life spans (T test *p* < 0.0001). B and C, *esg*-positive (*esg*^+^) cells (green) in control **B.,** and rapamycin treated **C.,** young (3 Days old) fly guts. **D.**-D′, esg^+^cells (green) and ISCs (by Delta) in control aging (20Days old) fly guts. **E.**-E′, esg^+^cells (green) and ISCs (by Dl) in rapamycin treated aging (20Days old) fly guts.**F.**, Quantification of midgut GFP cells in control and rapamycin treated 20Days old guts. Mean±SD is shown. *n* = 8-10 guts. **p < 0.01.

The addition of rapamycin in dose 200μM in the food extended *Drosophila* lifespan in our study (Figure 1A), which is similar to the previous report [1]. Whether rapamycin exerts an effect on lifespan extension by maintaining the intestinal homeostasis remains to be explored. To test this hypothesis, we first assessed the relationship between the proliferation rate of ISCs and lifespan in the aging guts in presence of rapamycin. We used a heat-inducible system in which *esg-Gal4* is combined with a temperature-sensitive Gal80 (TARGET system), and the flies were maintained at 25°C until the day before dissection and shifted to 29°C for 24h, to allow expression of the GFP in ISCs and EBs. Only GFP positive cells can show the ISC proliferation rates as *esg* is specifically expressed in ISCs and EBs whereas Delta specific expression is shown only by ISCs in intestine. We found that the proliferation of ISCs in the guts of young flies (3 days old), is maintained at low level in both control and rapamycin groups (Figure 1B–1C). Whereas in aging flies guts (20 Days old) there was a significant decrease in the number of GFP and Delta positive cells in rapamycin treated group when compared to the control group (Figure 1D–1F). The result showed that addition of rapamycin in the food can slow down the proliferation rate of ISCs in the aging guts and therefore can contribute towards the lifespan extension in *Drosophila.*

### Rapamycin slows down the intestinal barrier dysfunction and activates autophagy in the aging guts

**Figure 2:**
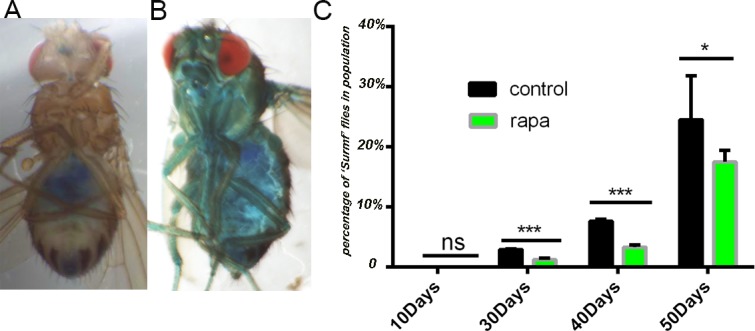
Rapamycin slows down the intestinal barrier dysfunction **A.**, A 10 days old fly after consuming a non-absorbed food dye (FD&C blue dye #1). The dye is restricted to the proboscis and digestive tract. **B.**, A 40 days old aged “Smurf” fly after consuming the same food dye. The blue dye is seen throughout the body due to loss of intestinal integrity. **C.**, Analysis of intestinal integrity as a function of age. In control flies, the percentage of “Smurf” flies in the population increases with age. Rapamycin pretreatment improves intestinal integrity in aged flies. (***p < 0.001, *n* >60 females for each genotype), Data are represented as mean ± SEM.

Flies with intestinal barrier dysfunction display increased expression of antimicrobial peptides (AMPs), impaired IIS and reduced metabolic stores compared with age-matched flies without intestinal barrier defects [14]. We tested the barrier dysfunction display to highlight the intestinal aging at the tissue level. Loss of intestinal integrity can be assayed in living flies by monitoring the presence of non-absorbed dye (FD&C blue no. 1) outside of the digestive tract post feeding [30]. As expected, we observed that in young flies (10 days old) the dye is restricted to the proboscis and digestive tract after feeding with FD&C blue no. 1 (Figure 2A), however, in aged flies (35 days old) we observed that a fraction of flies displayed a strikingly different phenotype. In these flies, the blue dye was clearly visible throughout the body after feeding; subsequently, these flies were referred to as “Smurf” flies (Figure 2B). In control flies, the percentage of “Smurf” flies in the population increases dramatically with age, from 0% at day 10 to 23% at day 50. Interestingly, flies upon rapamycin treatment retards the age-related onset of the “Smurf” phenotype (Figure 2C). Therefore, rapamycin improved intestinal integrity in aged flies, which is consistent with the delay in disruption of apical basal polarity in intestines from aging flies [14].

**Figure 3:**
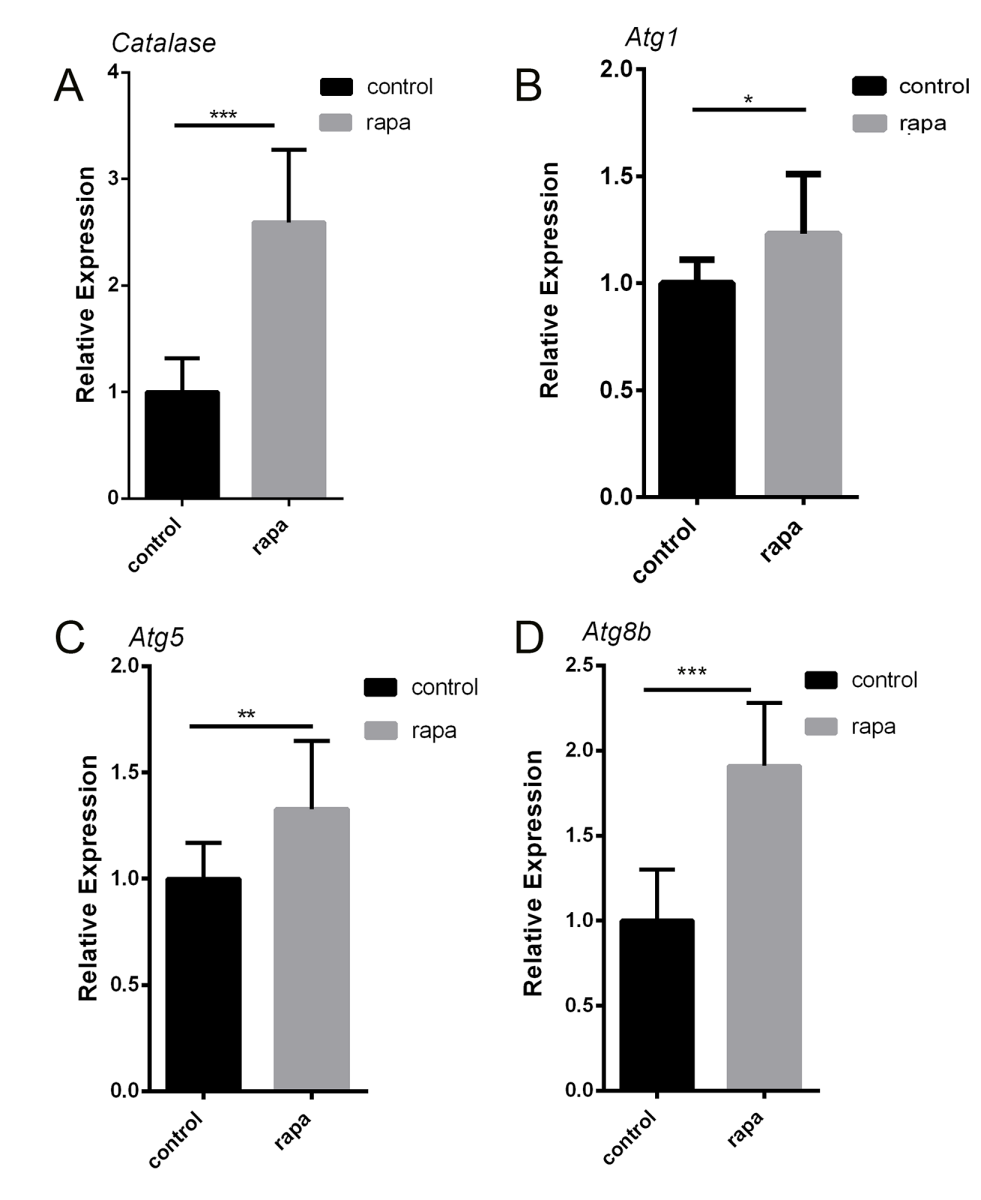
Rapamycin activating the autophagy in the aging guts **A.**, Intestinal expression of antioxidant gene *catalase* in 30 days old female fly guts. **B.**-**D.**, Expression levels of autophagy genes in 30 days old female fly guts. (*p < 0.05, ** *p* < 0.01,***p < 0.001, *n* = 3 of RNA extracted from 7 intestines/replicate).

It is previously reported that the intestinal stem cell population in the midgut of *catalase* mutant flies was increased compared to controls [31]. Catalase is a very important enzyme in protecting the cell from oxidative damage by reactive oxygen species (ROS). We also tested the *catalase* expression level in the aging guts and the result showed that the catalase is significantly over-expressed in rapamycin treated group (Figure 3A). This suggests that rapamycin can improve the intestinal capacity for antioxidants. We also assayed markers of autophagy in the intestine upon rapamycin treatment. Interestingly, we observed increased mRNA levels of *Atg1*, *Atg5*, and *Atg8b* in intestinal tissue (Figure 3B-3D), which indicates that rapamycin can induce autophagy and that may play a role in lifespan extension|.

### Effect of rapamycin treatment on FOXO associated genes in the intestine

**Figure 4:**
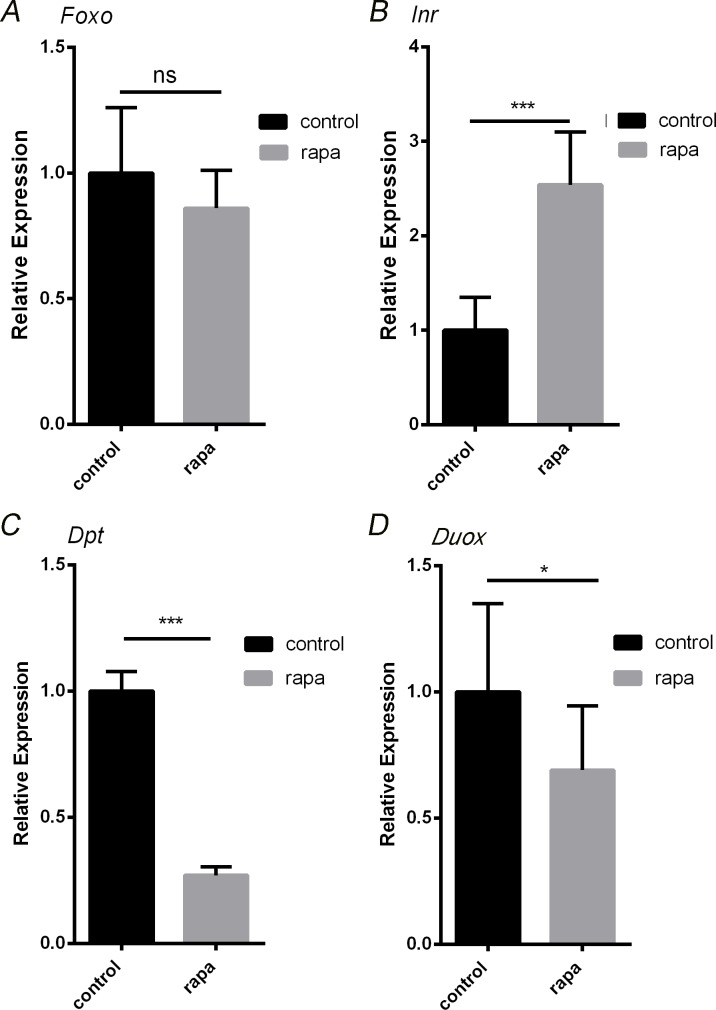
Rapamycin treatment impacts FOXO associated genes in the intestine **A.**, Expression levels of gene *Foxo* in intestines of 30 days old flies at control and rapamycin induction. **B.**, Expression levels of insulin receptor (*InR*) in intestines of 30 days old flies at control and rapamycin induction. **C.**, Expression levels of AMP gene *Dpt* in intestines of 30 days old flies at control and rapamycin induction. **D.**, *Duox* mRNA expression were quantified by RT-PCR in intestines of 30 days old flies at control and rapamycin induction. (***p < 0.001, *p < 0.05).

Rapamycin induces the phosphorylation of FOXO1 without any significant change in the RNA expression level. Rapamycin can elevate the expression levels of Insulin receptor (*Inr*) [32], which is present at the beginning of Insulin pathway and can repress FOXO phosphorylation by AKT1. When we checked the *Foxo* RNA expression level in aged guts by qRT-PCR, we didn't find any significant change (Figure 4A), whereas rapamycin treatment resulted in significantly elevated *Inr* expression (Figure 4B).

Intestinal barrier dysfunction display is always accompanied by increased AMPs expression level during the aging process. IIS-regulated *Foxo* can transcriptionally activate AMP genes through both Rel/NFkB-dependent and independent mechanisms [30-33]. For further uncovering the mechanism of rapamycin induced slow down of the intestinal aging, we detected the RNA expression level of *diptericin* (*dpt,* one of AMPs genes) in the aging guts. The result showed that, the *dpt* expression level in the aging intestinal tissue decreased four folds in rapamycin treatment group when compared to the control (Figure 4C). We also tested the dual oxidase (*Duox*) gene which is associated with ROS level and is a likely cause of age-related loss of epithelial homeostatis [23]. In aging fly intestine the *duox* expression level is significantly decreased when the rapamycin is added (Figure 4D), indicating that rapamycin may reduce the intestinal ROS accumulation in the aging flies, which means lower level of ROS accumulations are helpful in maintaining the epithelial homeostasis.

### Rapamycin treatment delayed the microbiota intestinal dysplasia by up-regulating the negative regulators of IMD/Rel pathway

**Figure 5:**
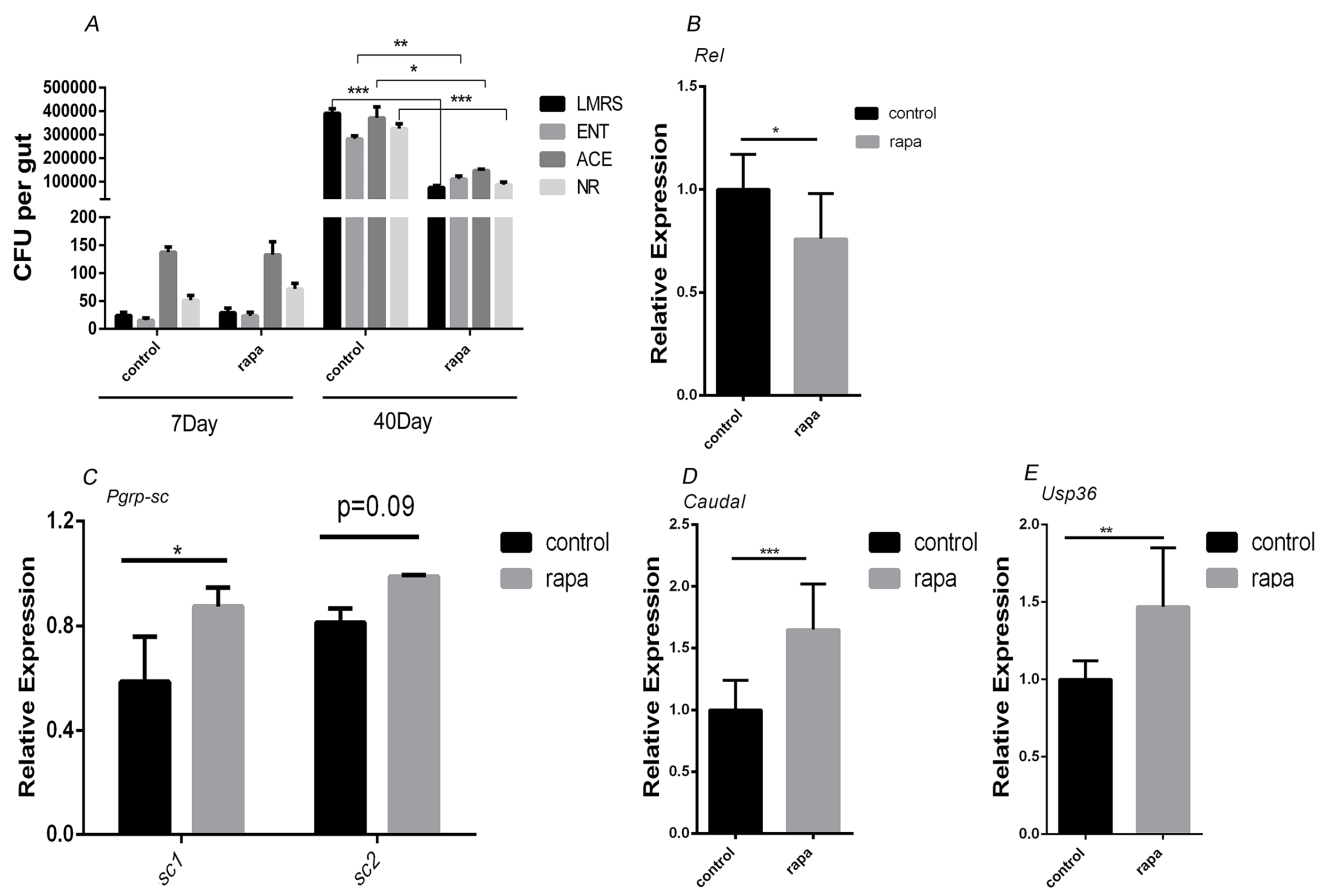
Rapamycin treatment delayed the commensal intestinal dysplasia by up-regulating negative regulators of IMD/Rel pathway **A.**, CFUs in intestinal extracts of control and rapamycin treated flies. Midgut homogenates from flies at 7 days or 40 days of age were plated on nutrient-rich medium (NR) or on selective plates allowing growth of *Lactobacilli* (LMRS), *Acetobacteria* (ACE), or *Enterobacteria* (ENT). **B.**, qRT-PCR for *rel* expression **C.**-**E.**, qRT-PCR for *pgrp-sc1*, *pgrp-sc2*, *caudal* and *usp36* expression (*n* = 3; *p < 0.05,**p < 0.01,***p < 0.001).

*Duox* is transcriptionally induced in ECs and activated in response to a microbial challenge [34]. We found that the *duox* expression level is reduced in the rapamycin treated aging guts (Figure 4D). We first confirmed that age-related dysplasia was associated with increased microbial loads in control flies. We quantified overall microbial load by measuring colony-forming units (CFUs) in dissected guts of young and aging flies, using selective plates to identify *Enterobacteriaceae*, *Lactobacillae*, *Acetobacteriaceae* and other bacteria growing on nutrient-rich medium. When compared to the control at 7 days of age, the number of CFUs in the gut of flies increased exponentially for all analyzed bacterial phylotypes at 40 days of age. While there was not any obvious gut size change observed after long-term rapamycin treatment, addition of rapamycin in the food can significantly reduce the number of CFUs for all analyzed bacterial phylotypes at 40 days of age (Figure 5A), indicating that rapamycin can delay the microbial expansion in the aging guts.

At the same time management of the commensal flora and innate immune responses to pathogens are achieved primarily in *Drosophila* ECs by activation of the immune deficiency (IMD/Relish) pathway, which activates the NFkB-like transcription factor Relish (*Rel*). We found that the *Rel* activity in the rapamycin treated gut is lower than the control fly gut (Figure 5B). In order to assess whether rapamycin delay the microbial expansion in the aging guts, we examined the gene expression of the negative regulators of IMD/Rel pathway, *Caudal*, *USP36*, *PGRPs-SC*, which are important in maintaining homeostasis. The result showed that they all are significantly up-regulated (Figure 5C–5E), suggesting that rapamycin can up-regulate the negative regulators of IMD/Rel pathway therefore keeping the immune homeostasis and delaying the microbial expansion in the aging guts.

## DISCUSSION

By using *Drosophila* as a model, we have shown that rapamycin can slow down the proliferation rate of intestinal stem cells (ISCs) in the aging guts; induce autophagy in the intestinal epithelium; affect the FOXO associated genes in intestine; and up-regulate the negative regulators of IMD/Rel pathway. The results uncovered that rapamycin can delay the intestinal aging by keeping the proliferation of the stem cells in control. Possibly it could be one of mechanisms underlying extension of lifespan in *Drosophila*.

Stem cell aging is a cause of organismal aging especially in high-turnover tissues such as animal intestine and dysregulation of such processes is likely to promote cancer or degenerative diseases. According to the previous reports, limited rate of ISC proliferation in the aging intestine can extend the lifespan [13-15, 30]. We hypothesize that rapamycin can slow the ISCs proliferation rates and ultimately promote longevity. Evidently ISCs become dysregulated in aging flies, over proliferate and produce a large number of cells that undergo incomplete differentiation [20]. This triggers intestinal dysplasia, which is characterized by loss of apico-basal organization of the epithelium and accumulation of mis-differentiated cells at the basement membrane [14]. In our results the number of ISCs and EB cells are significantly less in rapamycin treated aging guts in comparison to age-matched control flies. During aging the TORC1 hyperactivation leads to ISCs lost, but treatment with rapamycin in adult stage may help to maintain the ISCs [26].

A microbial challenge in the gut will induce the *duox* expression in ECs [34]. In our study we found that *duox* expression level is reduced in the rapamycin treated aged guts which indicates that rapamycin is delaying the microbial expansion during gut aging process. The age-related decline of proliferative homeostasis seems to be fundamentally a consequence of dysregulation of the interaction between the intestinal epithelium and the commensal bacterial population, resulting in commensal dysbiosis [15]. *Drosophila* lifespan is affected by intestinal dysplasia, and the dysplasia can be prevented by maintaining flies axenically. An age-related impairment in the ability to manage the intestinal microflora thus appears to be the underlying cause of intestinal dysplasia, limiting lifespan [15]

In the aging intestine of *Drosophila*, chronic activation of the transcription factor Foxo reduces expression of the IMD/Rel pathway negative regulators (PGRP-SC et al) [15]. Rapamycin inhibits the TOR pathway thereby the negative feedback loop is not effective, which activates the PI3K/Akt signaling pathway and up-regulates the IRS-1. The Akt1 can induce the phosphorylation of FOXO1 and prevent its entry to the nucleus, that can reduce the chronic activation of FOXO [35, 36]. In the aging guts this repression causes dysregulation of Rel/NFkB activity, resulting in commensal dysbiosis. We observed the *InR*, *caudal, usp36* and *pgrp-sc1*, are significantly up-regulated in the rapamycin treated aged guts. It seems that rapamycin may improve innate immune homeostasis to stabilize the gut microbiota that help to maintain the gut homeostasis and improve health and lifespan.

In the present study we found that the AMP gene *dpt* expression level is significantly decreased and the intestinal dysfunction is delayed in the aged flies gut. Intestinal barrier dysfunction plays a critical role in the etiology and/or mortality associated with many age-related diseases [37-41]. Flies with intestinal barrier dysfunction displayed increased expression of AMPs, impaired IIS and reduced metabolic stores compared to age-matched flies without intestinal barrier defects [14].

Here we have shown that rapamycin treatment preserves gut homeostasis during the *Drosophila* aging, which contributes to the healthspan and lifespan extension. Due to the high degree of evolutionary conservation in the mTOR pathway, we expect that the mechanism underlying rapamycin treatment for maintaining gut homeostasis could apply in other species as well. Future studies to investigate the effects of rapamycin in disease model systems may reveal potential common therapies for a wide range of age-related ailments.

## MATERIALS AND METHODS

### Fly stocks and husbandry

The wild-type stock Dahomey was collected in 1970 in Dahomey (now Benin) and has since been maintained in large population cages with overlapping generations on a 12L:12D cycle at 25°C. esgGal4, UAS-GFP, tub Gal80^ts^ (gift from N. Perrimon). All stocks were maintained and experiments were conducted at 25°C on a 12 hr:12 hr light:dark cycle at constant humidity using standard sugar/yeast/agar (SYA) media [28]. For all experiments, flies were reared at standard larval density and eclosed adults were collected over a 12 hr period. Flies were mated for 48 hr before sorting into single sexes.

### Lifespan analysis

Flies that eclosed over a 36 hour period were collected and allowed to mate for approximately 60 hours. Females were randomly allocated to the experimental food treatments and housed in plastic vials containing food at a density of 10 flies per vial, with 10 vials per condition (*n* = 100). Flies were transferred to a fresh food source 3 times per week, during which any deaths and censors were recorded. Rapamycin (Sigma) was dissolved in ethanol and added to SYA food at concentrations 200μm. For control food ethanol alone was added.

### Immunostaining and fluorescence microscopy

In all experiments, only the female posterior midgut was analyzed. The immunostaining of intestines was performed as previously described [29]. The primary antibodies used were mouse mAb anti-Dl (C594.9B, 1:50). All images were captured by a Zeiss LSM780 inverted confocal microscope and processed in Adobe Photoshop and Illustrator.

### Analysis of gene expression

Total RNA from 7 guts, were extracted using Trizol and cDNA was synthesized using Superscript II (Invitrogen). Real time PCR was performed in triplicates using SYBR Green, on Biorad IQ5 instrument and the following primer pairs were used.

(*Actin5C*-5′CTCGCCACTTGCGTTTACAGT, *Actin5C*-3′TCCATATCGTCCCAGTTGGTC.

*catalaes*-5′ATGCGGCTTCCAATCAGTTGAT, *catalaes*-3′CGAAGTGCGACATCTCATCCA. *Atg*1-5′GCCAGCTCCATCGAAAATAACC, *Atg*1-3′GCGGCGCAGCAGGCACAG.

*Atg*5-5′GCCCCTGCGACTTCACTATCC, *Atg5*-3′ CCATTAAATCGGCCAAACTCTTCT.

*Atg8b*-5′AATGTGATCCCACCGACATC, *Atg8b*-3′TTGAGCGAGTAGTGCCAATG.

*Dpt*-5′ GGCTTATCCGATGCCCGACG, *Dpt*-3′ TCTGTAGGTGTAGGTGCTTCCC.

*Duox*-5′ GTCGCACGCCAACCACAAGAGACT, *Duox*-3′CACGCGCAGCAGGATGTAAGGTTT.

*Foxo*-5′ TGTCGCTGCACAACCGCTTTATGA, *Foxo*-3′TTGCCGGAAATCGGGCGATAATTG.

*InR*-5′ CATCGGAAGGGAGGCGTAA, *InR*-3′CGTTTGCCTAATCGTCGAACA.

*Rel*-5′ ACAGCTACAGGAACTGCATCAGGAA, *Rel*-3′TCATCCTCCTCGAAGAACCTCACT.

*Pgrp-sc*2-5′ AACTACCTGAGCTACGCCGTGAT,

*Pgrp-sc2*-3′ AGCAGAGGTGAGGGTGTTGGTATT.

*Pgrp-sc1*-5′ CTATGTCGTCTCCAAGGCGGAGT,

*Pgrp-sc1*-3′ CGATCAGGAAGTTGTAGCCGATGT.

*cad*-5′ CGCCATCGAAGCCGCCATAC, *cad*-3′ ACCGCCGCCAAGGAGTGGCT.

*Usp36*-5′ GCCAAGAGCGGCGAGGACAC, *Usp*36-3′CAATTGGCCAGGGCGGGTATG.).

Fold changes in expression were calculated using the 2^−ΔΔCt^ method and normalized to *actin* (house keeping gene) levels. Results are average+/−standard deviation of at least 3 independent biological samples run in triplicates.

### Smurf assay

Unless stated otherwise, flies were aged on standard medium until the day of Smurf assay. Dyed medium was prepared using standard media with dyes added at a concentration of 2.5% (wt/vol). Blue dye no.1 was purchased from Sigma-Aldrich. Flies were maintained on dyed medium for 9 h. A fly was counted as a Smurf when dye coloration could be observed outside of the digestive tract. To calculate the SIR (Smurf increase rate), we plotted the average proportion of Smurfs per vial as a function of chronological age and defined the SIR as the slope of the calculated regression line [30].

### Selective plates for bacterial cultures

Selective plates were generated according to the following recipes:

*Acetobacteriaceae*: 25g/l D-mannitol, 5g/l yeast extract, 3g/l peptone, and 15g/l agar.

*Enterobacteriaceae*: 10g/l Tryptone, 1.5g/l yeast extract, 10g/l glucose, 5g/l sodium chloride, 12g/l agar.

*Lactobacilli* MRS agar: 70g/l BD Difco *Lactobacilli* MRS agar.

Nutrient Rich Broth: 23g/l BD Difco Nutrient agar.

All media were autoclaved at 121 degree for 15 min.
